# Microsaccade Characteristics in Neurological and Ophthalmic Disease

**DOI:** 10.3389/fneur.2018.00144

**Published:** 2018-03-13

**Authors:** Robert G. Alexander, Stephen L. Macknik, Susana Martinez-Conde

**Affiliations:** ^1^State University of New York (SUNY) Downstate Medical Center, Brooklyn, NY, United States

**Keywords:** microsaccades, fixational eye movements, square-wave jerks, Parkinson’s disease, progressive supranuclear palsy, amblyopia, strabismus, fixational saccades

## Abstract

Microsaccade research has recently reached a critical mass of studies that allows, for the first time, a comprehensive review of how microsaccadic dynamics change in neurological and ophthalmic disease. We discuss the various pathological conditions that affect microsaccades, their impact on microsaccadic and other fixational eye movement dynamics, and the incipient studies that point to microsaccadic features as potential indicators of differential and early diagnoses of multiple clinical conditions, from movement disorders to attention-deficit hyperactivity disorder to amblyopia. We propose that the objective assessment of fixational eye movement parameters may help refine differential diagnostics in neurological disease and assist in the evaluation of ongoing therapy regimes. In addition, determining the effects of ophthalmic disease on fixational eye movement features may help evaluate visual impairment in an objective manner, particularly in young patients or those experiencing communication difficulties.

## Introduction

When we attempt to fixate our gaze on a target, our eyes are never still, but produce small “fixational eye movements,” which include tremor, drift, and microsaccades. Microsaccades (also called fixational saccades) occur at a typical rate of 1–2 Hz. Converging research points to a saccadic generation continuum, which extends from the smallest fixational microsaccades to the largest exploratory saccades (1–5). Drift is a slow (typically less than 2°/s) motion that occurs between microsaccades and saccades, and travels in an erratic pattern that has been modeled as a random walk (6). Tremor (or ocular microtremor) occurs simultaneously with drift, during intersaccadic intervals. This is the smallest fixational eye movement, with amplitudes that approximate the width of a single photoreceptor and dominant frequencies between 70 and 103 Hz (averaging 84 Hz) (7, 8). Tremor studies are much scarcer than those centered on microsaccades and/or drift, due to the technical difficulties inherent to measuring this tiny motion (8, 9). Thus, we do not address tremor in this review.

Because we spend approximately 80% of our waking hours fixating our gaze [not only in a sustained way but also in transient fashion, between large saccades (10)], understanding fixational dynamics is critical to advance current knowledge of oculomotor and visual function. Fixational eye movement assessments may also help further our understanding of central and peripheral pathologies that result in impaired fixation.

Various neurological and ophthalmic disorders produce abnormal fixational eye movement patterns, with distinctive characteristics. Thus, establishing how neurological and ophthalmic disease affects fixational dynamics holds the potential to help in the early and differential diagnosis of such disorders, clarify their pathophysiology, and quantify their progression and response to treatment. Recent research efforts have set out to characterize fixational dynamics in a growing record of neurological and ophthalmological conditions, which we discuss in this review.

A classification of abnormal eye movements in different disorders of fixation was previously published [see Table 1 of Martinez-Conde (10)]. The intervening decade has seen an upsurge in fixational eye movement research, with an emphasis on microsaccade studies. In addition, cross-fertilization between fundamental and translational approaches to fixational dynamics has facilitated the identification of previously unknown links between (micro)saccadic eye movements and saccadic intrusions (the latter formerly relegated to the clinical literature).

Such recent developments have resulted in a critical mass of studies that allows us, for the first time, to offer a comprehensive review of how microsaccadic dynamics change in neurological and ophthalmic pathologies, from movement disorders to attention-deficit hyperactivity disorder (ADHD) to amblyopia.

## Microsaccades in Neurological Disease

The balance that fixational eye movement system must achieve in healthy oculomotor function is quite delicate: whereas insufficient eye motion can result in visual losses due to neural adaptation and visual fading, excessive eye motion leads to blurred and unstable vision. This fine calibration is disrupted in patients of various neurological and neurodegenerative disorders who display increased gaze instability during the attempt to fixate (11). Recent research efforts aimed to characterize such fixation instability—with an emphasis on the dynamic of microsaccades and drift—in neurological disease seek not only to improve early and differential diagnosis and help evaluate the efficacy of concurrent treatments but also to gain a deeper understanding of the pathophysiology and pathogenesis of such disorders.

### Microsaccades, Saccades, and Saccadic Intrusions in the Healthy Brain and in Neurological Disease

Converging evidence from physiological and behavioral studies conducted over the last decade has led to the current consensus that microsaccades and saccades—though previously considered as two different eye movement types—share a common oculomotor generator [(3); for review see Ref. (12)]. More recently, the proposal of a microsaccade-to-saccade continuum has been expanded to saccadic intrusions (3, 13, 14), defined as involuntary saccades that interrupt, or intrude on, precise fixation. Sacccadic intrusions are prevalent in certain neurodegenerative disorders, although healthy individuals also produce them. The most common saccadic intrusion is the square-wave jerk (SWJ), which consists of a small, horizontal saccade moving away from the fixation target, quickly followed by a corrective return saccade of equivalent amplitude and opposite direction. Though microsaccades and SWJs have most often been studied as two separate types of eye movements, recent work has put forward the notion that they, too, may be fundamentally the same kind of eye movement with different names (4, 5, 13, 14).

### Progressive Supranuclear Palsy (PSP) and Other Movement Disorders

**Figure 1:**
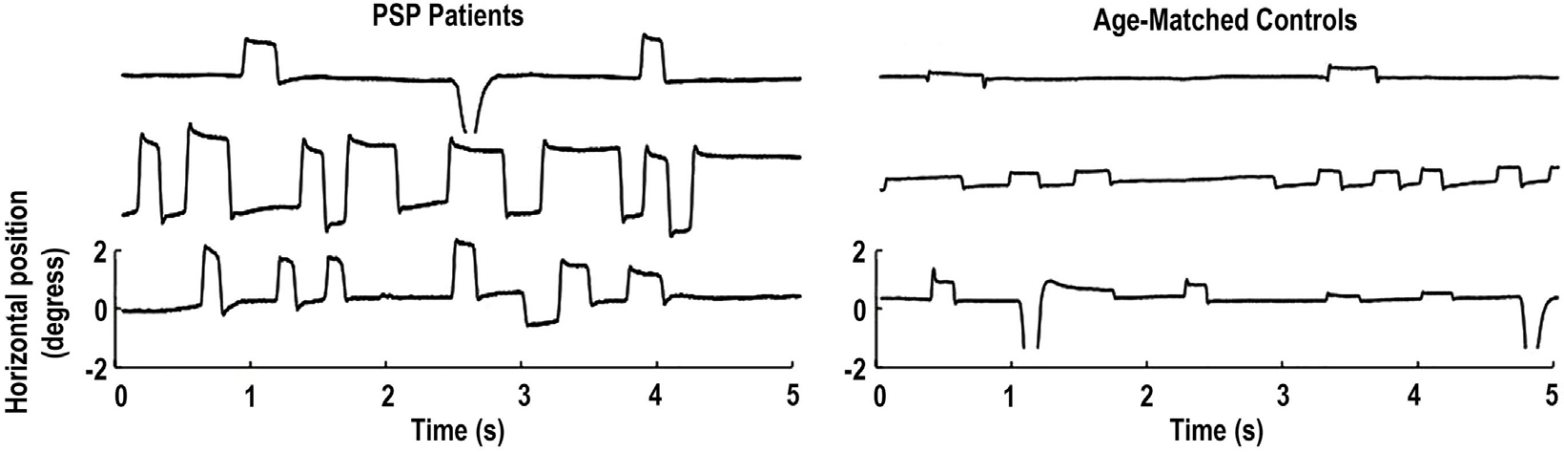
Square-wave jerks (SWJs) from three progressive supranuclear palsy (PSP) patients (left) and three age-matched controls (right). In both populations, (micro)saccades with amplitudes equal to or larger than half a degree of visual angle are paired as SWJs. Only the horizontal eye positions are shown. Modified from Otero-Millan et al. (14).

Pinnock and colleagues found larger and more frequent saccadic intrusions (including small intrusions due to microsaccades) in patients with Parkinson’s disease, multiple system atrophy, and PSP, than in healthy age-matched controls (15). Otero-Millan et al. subsequently set out to study the characteristics of microsaccades and SWJs in patients with PSP—a parkinsonian disorder that affects the basal ganglia, mesencephalon, and frontal lobe—in which SWJs are a distinctive clinical feature (14) (Figure 1).

**Figure 2:**
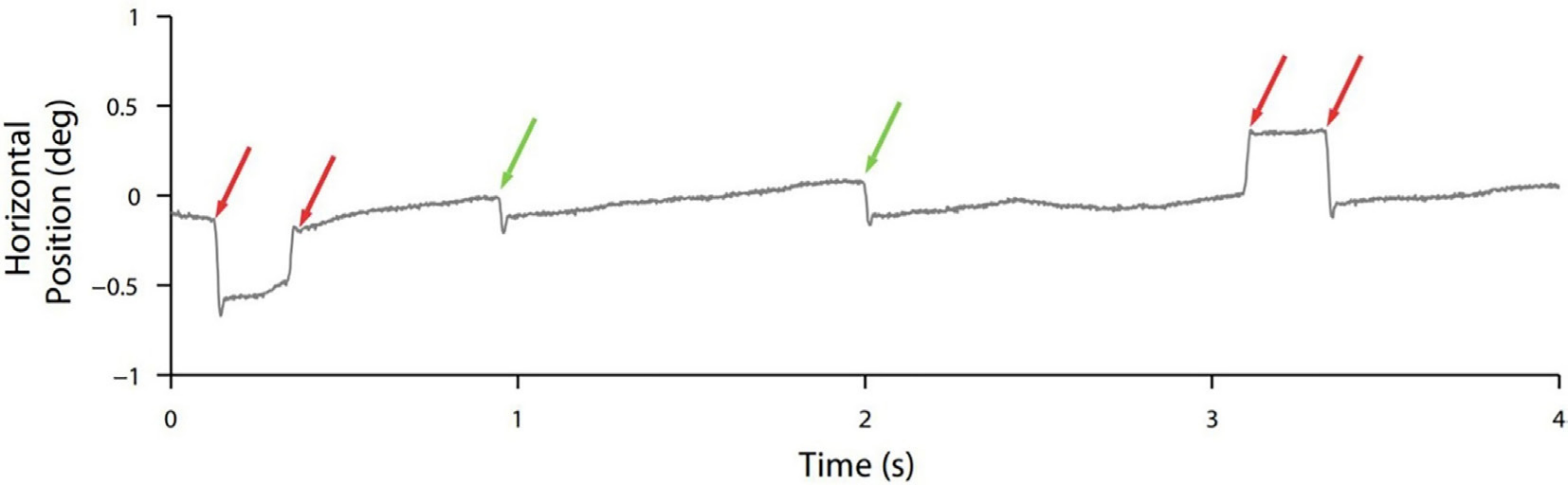
Square-wave coupling takes place for large but not small (micro)saccades. In this eye position trace from a healthy participant, red arrows point to pairs of larger microsaccades forming square-wave jerks; green arrows point to smaller unpaired microsaccades. From Otero-Millan et al. (14).

Though normal microsaccades were found to be rare in PSP, microsaccade magnitude was linked to SWJ coupling in both PSP patients and in healthy participants, with large microsaccades being more likely to trigger return saccades (forming SWJs) than small microsaccades (Figure 2). In addition, microsaccades and SWJs were slower in PSP patients than in controls, and they had a diminished vertical component, consistent with the vertical saccadic palsy that sets apart PSP from other parkinsonian patients. The results supported the hypothesis that a common mechanism may account for microsaccade and SWJ generation (13, 14) and explained how the position error from a large first saccade could serve as the trigger for the return saccade in SWJs produced by both PSP patients and healthy participants (16). The study concluded that the apparent distinction between microsaccades and SWJs could be due to two complementary mechanisms, underlying: (1) microsaccade production and (2) correction of gaze fixation errors due to oversized microsaccades (14). These two factors, combined, could explain square-wave coupling, both for microsaccade pairs in healthy subjects and for saccadic intrusions in neurological patients suffering from PSP, Parkinson’s disease, and other movement disorders, including multiple system atrophy, corticobasal syndrome, and spinocerebellar ataxia (5, 14).

### Mild Cognitive Impairment and Alzheimer’s Disease

Kapoula and colleagues recorded the eye movements of Alzheimer’s disease patients, patients with mild cognitive impairment, and healthy age-matched participants during the attempt to fixate. Whereas most microsaccadic features, including magnitude, velocity, duration, and intersaccadic intervals were equivalent across the three groups, oblique microsaccade directions were more prevalent in mild cognitive impairment and Alzheimer’s disease patients than in healthy participants (17). Layfield and colleagues wondered about potential links (positive or negative) between microsaccade dynamics and the amelioration of cognitive deficits in aging adults—following from targeted interventions known as “Speed Processing Training”—but found no relationship (18).

### Attention-Deficit Hyperactivity Disorder (ADHD)

The neural system that controls attention and the system that generates (micro) saccadic eye movements overlap extensively. Thus, multiple research studies have examined the connection between microsaccades, attention, and distractors [for review see Ref. (3)]. The superior colliculus, which plays a central role in (micro)saccade triggering, has moreover attracted recent interest as a potential site of dysfunction in ADHD (19, 20). A handful of studies have examined the connection between ADHD and gaze instability during fixation (21–23). Most recently, two studies have focused on the characteristics of microsaccades in individuals with ADHD (24) and ADHD traits (20). Fried et al. found a higher microsaccade rate in adult individuals with ADHD who were off medication than in control participants. Methylphenidate medication served to normalize microsaccade rates in the ADHD group. Panagiotidi and colleagues similarly found differing microsaccade rates in non-clinical participants with high and low levels of ADHD-like traits (20), assessed with the Adult ADHD Self-Report Scale (25). These combined results suggested that abnormal fixation behavior is a core deficit in ADHD, which could aid in the development of a biomarker for the disorder (20). Another recent study set out to investigate the impairment of temporal expectations in ADHD, by examining the inhibition of microsaccades prior to the onset of predicted stimuli. The data indicated decreased microsaccade inhibition in participants with ADHD than in controls, suggesting that microsaccade characterization may help enhance current understanding of the range of cognitive deficits that affect ADHD individuals (26).

### Autism Spectrum Disorder (ASD)

Recent research has found increased drift in autistic individuals (27). Microsaccade sizes and rates during fixation of a small target were comparable in ASD and neurotypical participants (27, 28), but those with ASD presented greater fixation instability, more microsaccades, and larger microsaccades when asked to maintain fixation on a blank screen with no target (28).

### Tourette Syndrome

A recent study found patients with Tourette syndrome to have reduced microsaccade amplitudes and increased intersaccadic intervals, along with increased fixation instability and drift velocities (29).

### Schizophrenia

Egaña and colleagues (30) found that previously reported decreased oculomotor function—in terms of decreased saccade and fixation rate—in schizophrenic patients (31) no longer differed from that of control participants once they included microsaccades in the analyses. In other words, schizophrenic patients made similar numbers of overall eye movements as healthy individuals, but produced fewer large, exploratory saccades to scan wide regions of the visual field. This study shows that fixational eye movement analyses in neurological and psychiatric disorders can be valuable not only to differentiate across populations but also to reveal previously unknown similarities between groups.

### Cerebral Palsy

Kozeis and colleagues proposed that microsaccadic impairment might complicate the acquisition of reading skills in children with cerebral palsy (32), but no studies to date have directly characterized fixational eye movements in this disorder.

### Hemianopia and Cortical Blindness

Hemianopia, or blindness in one-half of the visual field, can result from any lesion impairing post-chiasmatic central visual pathways. Reinhard and colleagues found microsaccadic distributions in hemianopic patients to be asymmetrical, with microsaccade directions biased toward the blind hemifield (33).

Gao and Sabel (34) subsequently investigated the characteristics of microsaccades in hemianopic stroke patients, to determine their potential relationship with visual performance and to assess how microsaccadic direction might be related to visual defect topography. They found that hemianopia resulted in enlarged microsaccades with impaired binocular conjugacy. Alterations of microsaccade dynamics worsened over time, being most prominent for older lesions. The data also revealed a microsaccade bias toward the seeing field, which was associated with faster reaction times to super-threshold visual stimuli in areas of residual vision, and suggested greater allocation of attention. Visual acuity was highest in patients with more binocular microsaccades and lower microsaccade velocities. The authors proposed that microsaccades may help compensate visual impairment in hemianopia and provide a basis for vision restoration and plasticity.

Blindsight is a rare phenomenon in which patients who have cortical blindness (due to lesions to the primary visual cortex) produce appropriate behavioral responses to visual stimuli they do not consciously see. Though no studies to date have systematically characterized microsaccadic properties in blindsighted patients, researchers in a recent case report studied microsaccadic inhibition (i.e., the transient suppression of microsaccade production after the presentation of a peripheral stimulus) in a patient who suffered from blindsight due to traumatic brain injury. The investigators observed that the patient’s microsaccade rates dropped briefly after the presentation of high- and low-contrast peripheral stimuli, in both the left (blind) and the right visual fields. In the case of low-contrast stimuli, the release from microsaccadic inhibition was slower in the blind field than in the sighted field, however (35).

### Short-Term Hypoxia

Di Stasi and colleagues found that saccadic velocity decreased and intersaccadic drift velocity increased, in connection with short-term hypobaric hypoxia in aviators. The finding that acute hypoxia diminishes eye stability, the authors proposed, may help to better understand the relationship between hypoxia episodes and central nervous system impairments (36).

## Microsaccades in Ophthalmic Disease

Vision and eye movements are intrinsically linked. Whereas it may seem intuitive to consider vision primarily in terms of its spatial characteristics, the process of seeing is a spatiotemporal one, where many timing features and constraints that impact our visual experience derive from the timing of eye movement production and targeting. Eye movements shape what we see, and our visual perception, in turn, affects the way we move our eyes. Ophthalmic disease, due to its deleterious effects on visual quality, tends to result in measurable abnormalities in eye movement properties, which extend to the fixational domain.

Because human beings are typically unaware of their fixational eye movements, studying their characteristics in ophthalmic disease may help evaluate the extent of a patient’s visual impairment in an objective manner—particularly in very young patients or those experiencing communication difficulties.

### Amblyopia and Strabismus

Most studies of fixational eye movements in ophthalmic disease to date have centered on amblyopia and strabismus. Amblyopia is defined as underdeveloped vision of one eye due to any condition that interferes with focusing during early childhood, including strabismus (in which the two eyes do not align correctly during fixation) and uncorrected refractive error (with anisometropia, or unequal refractive power in the two eyes).

Starting in the late 1970s, Ciuffreda and his colleagues conducted a series of pioneering studies on how amblyopia and strabismus affected fixation behavior (37–39). They found that, whereas amblyopic patients produced normal fixational eye movements during binocular fixation (and during monocular fixation with the fellow eye), monocular fixation with the amblyopic eye resulted in increased drift (whether or not strabismus was also present) (11, 37–40). If the amblyopia was due to strabismus, or in cases of alternating strabismus, this increase in drift was accompanied by sizable and frequent saccadic intrusions (37–39). By contrast, amblyopic fixation in dark-adaptation conditions was found to be normal or close to normal (41, 42).

**Figure 3:**
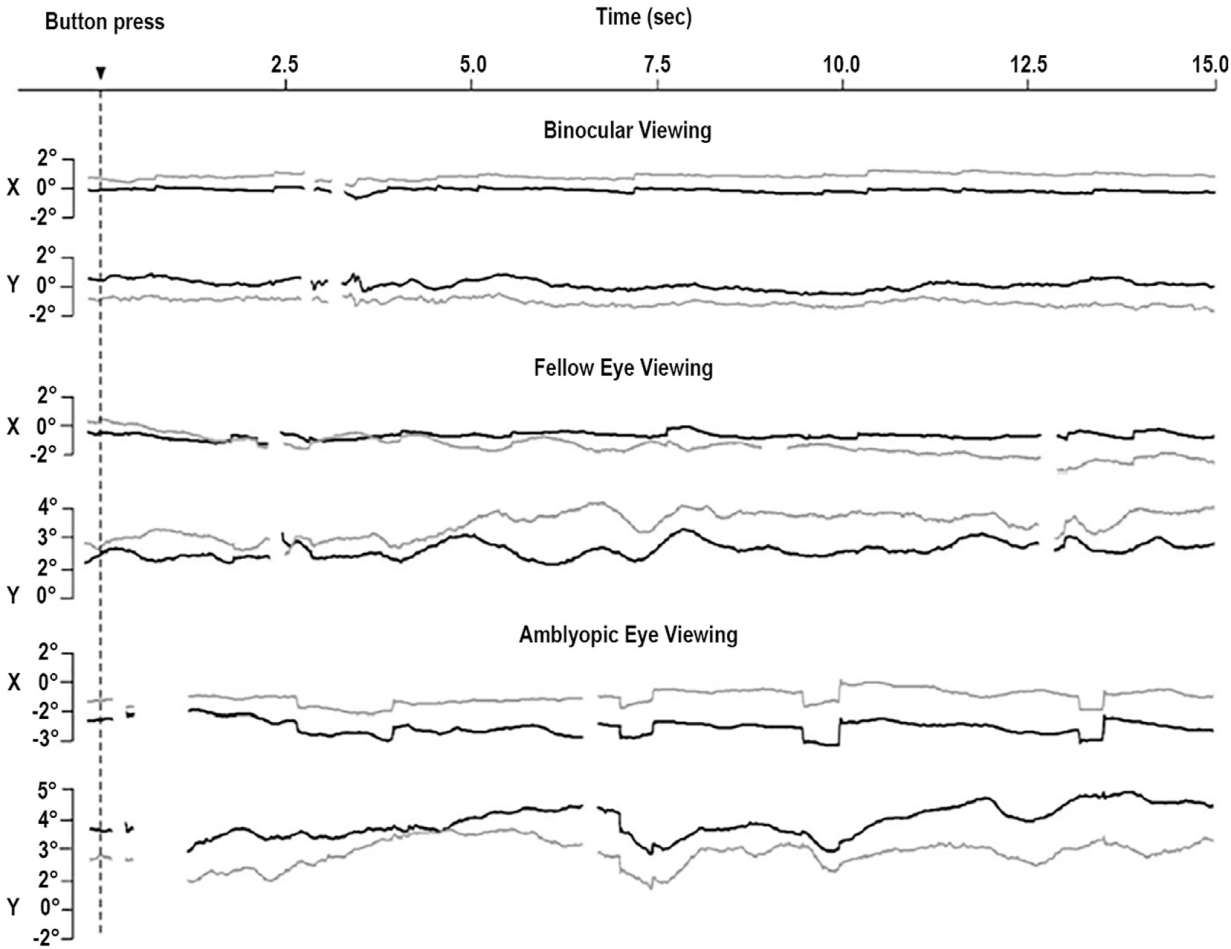
Eye movements of a patient with strabismic amblyopia. Horizontal (*X*) and vertical (*Y*) positions are plotted for the left eye (gray, amblyopic eye) and the right eye (black, fellow eye). Amblyopic eye viewing results in larger microsaccades in both eyes. Monocular viewing with the fellow eye is tied to increased instability in the amblyopic eye. Monocular viewing with the amblyopic eye produces increased instability in the two eyes. From González et al. (45).

More recently, Shi and colleagues found less frequent but larger microsaccades during monocular fixation with the amblyopic eye than with the fellow eye (43) and proposed that the objective evaluation of oculomotor function in amblyopia includes a microsaccade assessment. Otero-Millan et al. (44) noted that microsaccades produced during normal binocular fixation of large targets have similar features to those reported by Shi et al. during monocular fixation with the amblyopic eye, which might indicate a common lack of fixation precision in both scenarios. This possibility is consistent with work finding reduced fixation stability in the amblyopic eye, as compared to the fellow eye and to binocular viewing (45) (Figure 3).

**Figure 4:**
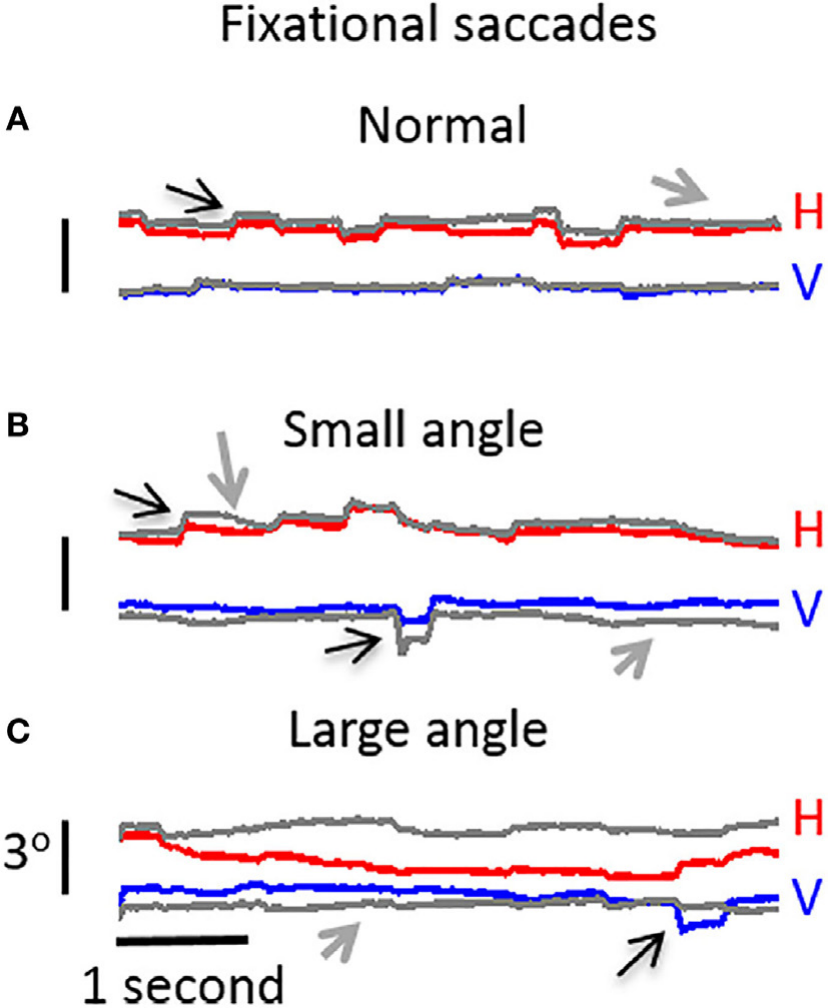
Visual fixation of a target for 5 s, under monocular viewing conditions. **(A)** Participant with normal vision: both microsaccades and intersaccadic drift are conjugate. **(B)** Participant with small-angle strabismus and stereopsis present: disconjugancy across the two eyes is mild for both microsaccades and drift. **(C)** Participant with large-angle strabismus and absent stereopsis: disconjugancy is pronounced for both microsaccades and drift. **(A–C)** Horizontal (red: right eye, gray: left eye) and vertical (blue: right eye, gray: left eye) eye positions are presented. Black arrows indicate microsaccades, and gray arrows represent intersaccadic drift. From Ghasia et al. (46).

Ghasia et al. found that fixational saccades and ocular drift were more disconjugate for patients with strabismus than for control participants. This disconjugacy was greater for patients with large-angle strabismus and impaired stereopsis (as a result of the misalignment of their eyes) than for patients with small-angle strabismus and preserved stereopsis (Figure 4). This study also found that drift was faster in patients with strabismus than in control subjects (46).

Fixation stability, usually measured as the eye position dispersion [i.e., bivariate contour elliptical area (BCEA)] during the attempt to fixate, combines the effects of microsaccades and drifts, without making a distinction between the two types of eye motion (44). Subramanian et al. (47) found that the BCEA was larger in the amblyopic eye than in the fellow eye, especially along the horizontal axis, and that patients with larger BCEAs tend to have lower visual acuities.

Increased drift and decreased microsaccade production in severe amblyopia may lead to perceptual fading of large portions of the visual field, including the “small fixation spot, small and large acuity targets, and even portions of the laboratory” during monocular fixation with the amblyopic eye (37, 48–50). One patient reportedly “made saccades to revive the faded or blanked-out portions” of the image in such situations (37), suggesting that visual fading in amblyopia might be related to ordinary Troxler fading [i.e., the kind of visual fading that normally sighted individuals can experience during fixation in the absence of microsaccades (10, 51)].

Increased drift in amblyopia could also produce lower visual acuity—and increased variability in visual acuity measurements—by shifting retinal images to more eccentric positions (39, 52). Such links exemplify the tight bond between the motor and sensory aspects of fixational eye movements (44).

Aiming to increase fixation stability for the amblyopic eye (and thus produce bifoveal fixation), Raveendran et al. (53) decreased the contrast of the image viewed by the fellow eye until it was equivalent to the contrast perceived by the amblyopic eye. Fixation stability in the amblyopic eye improved as a result, but bifoveal fixation was nevertheless temporary (due to the amblyopic eye drifting away from foveal alignment).

Loudon and colleagues used a binocularity score to assess how well subjects fixated a target with both eyes and proposed that the presence of fixation instability can help detect amblyopia at an early age (when it otherwise goes undetected up to a third of the time) (54).

Successful orthoptics therapy tends to normalize fixational eye movements in amblyopia [though not all oculomotor and visual functions may improve concurrently (52, 55)]. Thus, Ciuffreda and colleagues have proposed that amblyopic therapies should not be interrupted when patients achieve normal visual acuity and centralized fixation, but should continue until they produced normal or stabilized fixational eye movements. Because critical periods for some aspects of oculomotor plasticity may extend into adulthood, lack of fixational eye movement normalization in amblyopic patients could be a factor in their reverting to the pre-treatment condition once therapy is discontinued (52). Thus, fixational eye movement assessments may help establish the optimal duration of treatments.

### Central Scotoma due to Macular Disease or Dysfunction

**Figure 5:**
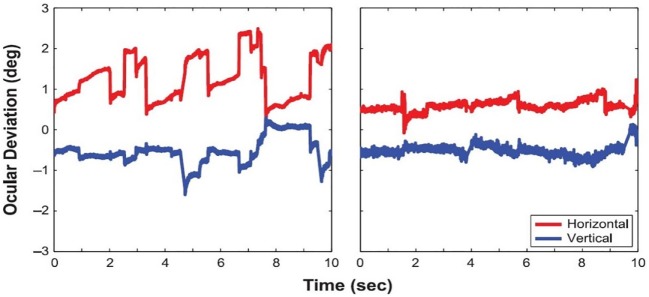
Eye positions during fixation in a patient with macular disease (left) and a healthy control subject (right). From Kumar and Chung (57).

Macular scotomas, and other pathologies producing prolonged monocular visual deprivation, have also been connected to increased drift, with comparable characteristics to drift in amblyopes (11, 56). A recent study by Kumar and Chung (57) found that patients with macular disease presented not only increased drift amplitudes but also larger microsaccadic amplitudes (Figure 5) than healthy subjects, without a corresponding change in microsaccade rate. The authors concluded that an increase in drift and microsaccade amplitudes—as opposed to changes in velocity or rate—is the strongest predictor of overall fixation instability in macular disease. Moller et al. previously found, in a group of diabetic maculopathy patients, that microsaccade magnitude increased as visual acuity decreased (58). A more recent study set out to determine if saccades in an eye affected by diabetic maculopathy were influenced by the other eye during binocular fixation. The results revealed that microsaccades during monocular fixation with the eye most affected by macular edema were larger, more frequent, and involved a larger retinal area than those produced during binocular fixation. A significant negative correlation was found between area of fixation and visual acuity during monocular, but not binocular, fixation. The authors concluded that binocular fixation can reduce the fixation area and microsaccade amplitude in the “worst eye” of diabetic maculopathy patients and advised that microsaccades in diabetic maculopathy are studied during monocular fixation (59).

### Myopia

Recent work found increased microsaccade amplitude—without a corresponding change in microsaccade velocity or microsaccade rate—in myopic individuals (60). As the severity of uncorrected refractive error increased, so did the sizes of microsaccades. This suggested that the control of microsaccade amplitude relies on the precision of visual information on the fovea, with blurred information leading to fixational instability.

### Retinal Implants

Here, we discuss current efforts to characterize fixational eye movements, including microsaccades, in patients with subretinal implants. Because subretinal implants are placed below the retina (unlike epiretinal implants such as the Argus II, where an external camera is used to capture an image), the eye movements of implanted patients have the potential to affect—and be affected by—their visual perception.

In recent research, patients with a subretinal Alpha IMS scanned their visual field to locate a fixation target that appeared at random locations. Upon target fixation, the patients produced fixational eye movements—including microsaccades (with and without square-wave coupling) and drifts—that were analogous to those produced by control participants (61). A previous study by the same group made similar observations (62). The properties of (micro)saccades moreover depended on the shape of the stimulus being viewed. For instance, both patients and control participants made more horizontal eye movements when viewing a rectangle than when viewing a square (61). These data suggest that (micro)saccadic dynamics might help provide an objective measure of the success of an implant, especially in situations where subjective reports are questionable or inviable: if eye movements characteristics change in response to changes in the stimulus, it would indicate that visual inputs have been processed appropriately (61).

One limitation of subretinal implants such as the Alpha IMS is that visible stimuli typically fade from perception within seconds (61–63). Understanding the relationship between fixational eye movement dynamics and image fading in prosthetic vision may prove key to improving future subretinal implants. Microsaccades have been shown to counteract, and to help prevent, perceptual fading in natural vision (51, 64–68). Similarly, microsaccade occurrence has been connected to fading prevention in patients with subretinal implants (61). It has also been proposed that the characterization of microsaccade patterns in patients could help fine-tune the frequency of stimulation that results in optimal visibility in specific individuals. That is, depending on a particular observer’s fixational eye movement patterns, he/she may need higher or lower stimulation frequencies to maintain visibility (69). Future research may investigate the translational value of this potential relationship.

It remains currently unknown why visual fading is more severe in patients with prosthetic implants than in healthy observers. One possibility is that fixational eye movements counteract and prevent perceptual fading less effectively in implanted patients than in natural vision (70). Recent modeling work has suggested that increased fading in prosthetic vision might be due to a lack of OFF responses and to lower contrast sensitivity than in natural vision (71); thus, the quality of the visual input may be too low for eye movements to refresh retinal images effectively. Fading may also be more or less prevalent depending on the size of electrodes used: stimulation from a single electrode may affect such a large visual region that even when microsaccades shift the stimulus to adjacent electrodes, they may not significantly change the activated neurons (70, 72).

## Conclusion

We have reviewed the characteristics of fixational eye movements in neurological and ophthalmic disease, with an emphasis on microsaccades. Though studies addressing microsaccadic impairments in patient populations remain relatively scarce, this has recently become an area of active inquiry, with valuable implications for both clinical and basic research (3). Converging studies have made significant headway vis-à-vis the potential of microsaccade and other fixational eye movement dysfunctions as indicators of ongoing pathologies beyond the oculomotor realm. Thus, the objective assessment of fixational eye movement parameters may help refine differential diagnostics and assist in the evaluation of ongoing therapy regimes (i.e., successful treatments should result in the normalization of previously impaired fixational eye movements). These measures will also help refine current understanding of the pathogenesis of neural disease, as well as place constraints on—and guide the development of—future saccadic generation models, especially with regards to the relationship of (micro)saccades to saccadic intrusions in neurological disease.

## Author Contributions

SM-C, RGA, and SLM wrote and edited the manuscript. SM-C and RGA conducted the literature review.

## Conflict of Interest Statement

The authors declare that the research was conducted in the absence of any commercial or financial relationships that could be construed as a potential conflict of interest.
